# Angiotensin II-superoxide-NFκB signaling and aortic baroreceptor dysfunction in chronic heart failure

**DOI:** 10.3389/fnins.2015.00382

**Published:** 2015-10-16

**Authors:** Dongze Zhang, Robert L. Muelleman, Yu-Long Li

**Affiliations:** Department of Emergency Medicine, University of Nebraska Medical CenterOmaha, NE, USA

**Keywords:** angiotensin II, baroreflex, baroreceptor, heart failure, nodose ganglion, nuclear factor κB, sodium channel, superoxide

## Abstract

Chronic heart failure (CHF) affects approximately 5.7 million people in the United States. Increasing evidence from both clinical and experimental studies indicates that the sensitivity of arterial baroreflex is blunted in the CHF state, which is a predictive risk factor for sudden cardiac death. Normally, the arterial baroreflex regulates blood pressure and heart rate through sensing mechanical alteration of arterial vascular walls by baroreceptor terminals in the aortic arch and carotid sinus. There are aortic baroreceptor neurons in the nodose ganglion (NG), which serve as the main afferent component of the arterial baroreflex. Functional changes of baroreceptor neurons are involved in the arterial baroreflex dysfunction in CHF. In the CHF state, circulating angiotensin II (Ang II) and local Ang II concentration in the NG are elevated, and AT1R mRNA and protein are overexpressed in the NG. Additionally, Ang II-superoxide-NFκB signaling pathway regulates the neuronal excitability of aortic baroreceptors through influencing the expression and activation of Na_v_ channels in aortic baroreceptors, and subsequently causes the impairment of the arterial baroreflex in CHF. These new findings provide a basis for potential pharmacological interventions for the improvement of the arterial baroreflex sensitivity in the CHF state. This review summarizes the mechanisms responsible for the arterial baroreflex dysfunction in CHF.

## Introduction

Chronic heart failure (CHF), the most common type of heart diseases, affects approximately 5.7 million people in the United States. During the past several decades, about 870,000 new cases were diagnosed and more than 58,309 individuals died from CHF in the United States each year (Mozaffarian et al., 2015). CHF is characterized by autonomic dysfunction, including withdraw of the parasympathetic tone and overactivation of the sympathetic tone (Creager et al., 1986; Saul et al., 1988; Porter et al., 1990), which is closely related to mortality in patients with CHF (Gronda et al., 2014). Impairment of the baroreflex sensitivity is directly associated with this autonomic dysfunction (Creager, 1992). Many studies have demonstrated that the arterial baroreflex sensitivity is attenuated in both clinical and experimental CHF (White, 1981; Floras, 1993; Frenneaux, 2004; Pinna et al., 2005; Ruttanaumpawan et al., 2008), which is a predictive risk factor for sudden cardiac death (Kleiger et al., 1987) and is associated with mortality of CHF (Nolan et al., 1998; Cygankiewicz et al., 2008; Boogers et al., 2011; Hauptman et al., 2012). Although the precise mechanisms responsible for blunted arterial baroreflex in the CHF state are not fully understood, activation of the arterial baroreflex (as a long-term therapeutic approach) has been shown to have numerous beneficial effects on CHF treatments in both clinical and experimental studies (Zucker et al., 2007; Sabbah, 2012; Doumas et al., 2014; Gronda et al., 2014; Halbach et al., 2014; Iliescu et al., 2014; Liao et al., 2014; Madershahian et al., 2014; Schmidli et al., 2014).

In general, the arterial baroreflex is a homeostatic mechanism by which arterial blood pressure and heart rate are changed through sensing the alteration of tension in arterial vascular walls. The arterial baroreflex arc is composed mainly of an afferent component, a central neural component, and autonomic neuroeffector components. Arterial baroreceptors are the main afferent component of arterial baroreflex arc, which are sensors located in the arterial blood vessels including aortic baroreceptor neurons in the NG and carotid baroreceptor neurons in the petrosal ganglion. They sense the mechanical alteration of arterial vascular walls through the baroreceptor terminals and increase the excitation of these afferent baroreceptors. This excitatory signal is integrated in the baroreceptors by local endogenous substances, and then the sensory information is communicated to the dorsal medial nucleus tractus solitary (NTS). The NTS in the brain recognizes the information from the baroreceptors and finally evokes the parasympathoexcitatory and sympathoinhibitory responses (Czachurski et al., 1982; Spyer et al., 1997; Benarroch, 2008). Each component of the arterial baroreflex arc might be involved in the impairment of the baroreflex function in CHF. It has been demonstrated that central autonomic pathways and neuroeffectors contribute to baroreflex dysfunction in CHF (Zucker et al., 1995; Zucker and Liu, 2000; Pan et al., 2007; Schultz, 2009; Wang et al., 2014). However, the impairment of arterial baroreceptors also is a major obstacle to influence the arterial baroreflex function in CHF (Dibner-Dunlap and Thames, 1989; Wang et al., 1990, 1991a,b; Rondon et al., 2006). Indeed, recent studies have shown that the functional impairment of aortic baroreceptor neurons located in the NG is involved in the baroreflex dysfunction in CHF (Tu et al., 2010; Zhang et al., 2014). In this brief review, therefore, we will focus mainly on our work to discuss the contribution of aortic baroreceptors in blunted arterial baroreflex in CHF, especially the possible cellular and molecular mechanisms responsible for the alteration of aortic baroreceptors.

## Voltage-gated sodium (Na_v_) channel remodeling in aortic baroreceptors contributes to blunted baroreflex sensitivity in CHF

Based on the fact that CHF is a chronic, progressive cardiovascular disease, it is possible that morphological changes and/or functional alterations in aortic baroreceptor neurons might be involved in the impaired baroreflex sensitivity in CHF. Morphological data from our published study have shown that both total neuron number and ratio of A-type/C-type neurons in the NG have no significant difference between CHF and sham rats (Tu et al., 2010). Using a dog model of CHF, Wang, et al. also measured the density of A-type and C-type nerve fibers in the carotid sinus and found that there is no change in A-/C-fiber ratio and total fiber density in CHF dogs, compared to sham dogs (Wang et al., 1996). These results from the morphological measurement support the view that cellular and molecular changes rather than morphological alterations in aortic baroreceptors could be involved in the arterial baroreflex dysfunction in the CHF state.

It is well-established that voltage-gated ion channels (e.g., sodium, calcium, and potassium voltage-gated channels) exist in the transmembrane of excitable cells, and these types of ion channels can affect cell excitability including neuronal excitation. Using path-clamp and molecular biological techniques, all major subtypes of sodium, calcium, and potassium voltage-gated channels have been functionally characterized in NG neurons including aortic baroreceptor neurons. These major subtypes of voltage-gated ion channels include: (1) tetrodotoxin (TTX)-sensitive and TTX-resistant Na_v_ channels; (2) N-type, L-type, T-type, and R-type calcium voltage-gated channels; (3) 4-aminopyridine-sensitive, tetraethylammonium-sensitive, and calcium-activated potassium voltage-gated channels (Li et al., 1998; Lancaster et al., 2002; Schild and Kunze, 2012; Tatalovic et al., 2012; Xu et al., 2015).

Na_v_ channels are responsible for initiation and propagation of the action potential in neurons including primary viscerosensory neurons (Yu and Catterall, 2003; Ritter et al., 2009). Thus, far, nine α-subunits (Na_v_1.1–1.9) of Na_v_ channels have been functionally characterized. Each Na_v_ channel subunit has particular tissue localization, consistent with a distinct role for each Na_v_ channel subunit in mammalian physiology. In general, Na_v_ channels in primary afferent neurons are separated into TTX-sensitive Na_v_ channels and TTX-resistant Na_v_ channels. TTX-sensitive Na_v_ channels are characterized by low activation threshold, fast activating and inactivating Na_v_ channels, which include Na_v_ 1.1, Na_v_ 1.2, Na_v_ 1.3, Na_v_ 1.4, Na_v_ 1.6, and Na_v_ 1.7 channels. TTX resistant Na_v_ channels are characterized by high activation threshold, slow activating, and inactivating Na_v_ channels, which include Na_v_ 1.5, Na_v_ 1.8, and Na_v_ 1.9 channels (Waxman et al., 1999; Yu and Catterall, 2003; Catterall et al., 2005). Based on the sensitivity of neurons to TTX, aortic baroreceptor neurons in the NG could be separated into A-type and C-type neurons (Schild and Kunze, 1997). TTX totally blocks Na_v_ currents in A-type aortic baroreceptor neurons, whereas TTX partially inhibits Na_v_ currents in C-type aortic baroreceptor neurons. TTX-blocked Na_v_ currents are defined as TTX-sensitive Na_v_ currents and remained Na_v_ currents are defined as TTX-resistant Na_v_ currents. Although Na_v_ 1.7, Na_v_ 1.8, and Na_v_ 1.9 channels are abundantly expressed in the NG (Waxman et al., 1999; Baker and Wood, 2001; Cummins et al., 2007; Kwong et al., 2008; Tu et al., 2010), their distribution in the NG is different. TTX-sensitive Na_v_ 1.7 channels are located in the cell transmembrane of both A-type and C-type nodose neurons, while TTX-resistant Na_v_ 1.8 and Na_v_ 1.9 channels are only expressed in the cell transmembrane of C-type nodose neurons (Tu et al., 2010).

Although the correlation of voltage-gated Na_v_ channels with initiation of the action potential and propagation of the neuronal discharge in primary sensory neurons has been discussed in many studies (Schild et al., 1994; Yoshida, 1994; Schild and Kunze, 1997; Matsutomi et al., 2006; Patrick Harty and Waxman, 2007), there is little known about the role of Na_v_ channels in determining the activity of baroreceptor neurons and arterial baroreflex, especially in the CHF state. One study from Shen et al. has shown that intravenous administration of Na_v_ channel enhancer restores the blunted baroreflex sensitivity in conscious dogs with CHF (Shen et al., 2005). Therefore, the alteration of Na_v_ channels is closely associated with blunted baroreflex sensitivity in the CHF state. Real-time RT-PCR, western blot, and immunofluorescent staining data in our study have demonstrated that the expression (mRNA and protein) of Na_v_ 1.7, Na_v_ 1.8, and Na_v_ 1.9 channels is decreased in the NG from CHF rats (Tu et al., 2010). Additionally, patch-clamp data have also shown that densities of both TTX-sensitive and TTX-resistant Na_v_ currents recorded in isolated aortic baroreceptor neurons are reduced in CHF rats. Furthermore, the suppression of cell excitability is observed in aortic baroreceptor neurons from CHF rats. When a Na_v_ channel activator, rATX II was administered in aortic baroreceptor neurons from CHF rats, it significantly restored CHF-decreased Na_v_ current density and cell excitability of aortic baroreceptor neurons (Tu et al., 2010). Therefore, the involvement of reduced Na_v_ channels in the aortic baroreceptor dysfunction in the CHF state is further evidenced by our study mentioned above (Tu et al., 2010).

To evaluate the role of aortic baroreceptors in the arterial baroreflex in the CHF state, we employed two methods. One method is to examine the changes in blood pressure and heart rate when the aortic depressor nerve is electrically stimulated. There are three advantages in this method for measurement of the baroreflex function. Firstly, rat aortic depressor nerves do not contain functional chemoreceptor afferent fibers for the generation of arterial chemoreflex, which means that there are only baroreceptor afferent fibers in the rat aortic depressor nerve to convey the electrical signal to the central nervous system (Sapru et al., 1981; Fan et al., 1996; Kobayashi et al., 1999). Secondly, a directly electrical stimulation of the rat aortic depressor nerve can bypass aortic depressor nerve terminals and aortic arterial vascular walls to induce the arterial baroreflex. Thirdly, by varying the frequency of electrical stimulus, reflex responses to activating A-type and C-type afferent fibers can be differentiated. However, a disadvantage of this technique is that a physiological substrate for the aortic baroreceptor activation is not represented. Another method is to measure reflex changes in heart rate and cardiac sympathetic nerve activity in response to changes in arterial blood pressure. The advantage for this method is that a physiological stimulation (changes in arterial blood pressure) is used to activate the arterial baroreflex. A major limitation in this approach is that possible alterations in the mechanotransduction process at the barosensory nerve terminal and the arterial vascular elasticity may also play a role in the arterial baroreflex function. Our previous *in vivo* studies have demonstrated that the arterial baroreflex is significantly depressed in CHF rats whatever electrical stimulation of the aortic depressor nerve or change in the arterial blood pressure is used to induce the arterial baroreflex (Tu et al., 2010; Zhang et al., 2014). Additionally, baroreceptor nerve stimulation-induced baroreflex sensitivity was markedly improved in CHF rats when the NG was treated by rATX II (a Na_v_ channel activator) (Tu et al., 2010). However, the local treatment of rATX II did not normalize the Na_v_ current density and neuronal excitability of aortic baroreceptors, and arterial baroreflex sensitivity in CHF rats toward the level seen in sham rats, suggesting that other mechanisms might be involved in this process. In physiological and pathophysiological conditions, acute changes of the ion channel kinetics and chronic alterations of the ion channel expression are two major factors to modulate the ion channel function. Based on the inability of rATX to improve the expression of Na_v_ channels, we consider that low level of Na_v_ channel expression in the NG from CHF rats might explain the above results. So far we cannot identify the contribution of each Na_v_ channel subunit to the cell excitability of aortic baroreceptor neurons and baroreflex sensitivity, because no specific Na_v_ channel activators are available for Na_v_ 1.7, Na_v_ 1.8, and Na_v_ 1.9 channels. These experimental results indicate that the remodeling of Na_v_ channels including the lower expression of Na_v_ channels and the decrease of Na_v_ currents could reduce the neuronal excitability of aortic baroreceptors and induce resultant impairment of the arterial baroreflex sensitivity in the CHF state.

Currently, there is no information available about the changes of voltage gated-calcium channels and potassium channels in aortic baroreceptor neurons in the CHF state. Therefore, we cannot rule out the involvement of these ion channels in the alteration of aortic baroreceptors and the arterial baroreflex dysfunction in CHF.

## Mitochondria-derived superoxide overproduction mediates the decreased Na_v_ currents and cell excitability in baroreceptor neurons in CHF

The mitochondrial electron transport chain contains several mitochondrial complex enzymes, which constitutes the main source of superoxide in physiological and pathophysiological conditions (McCord, 1993; Cadenas and Davies, 2000; Turrens, 2003; Balaban et al., 2005; Adam-Vizi and Chinopoulos, 2006; Murphy, 2009). Under physiological conditions, the mitochondrial electron transport chain transfers electrons to molecular oxygen for ATP production. Only a tiny leakage of electrons (1–2%) from the mitochondrial electron transport chain produces a low level of superoxide (McCord, 1993; Cadenas and Davies, 2000). The low level of superoxide is essential for normal cellular metabolism (Fattman et al., 2003). However, in pathophysiological conditions, the inhibition of mitochondrial complex enzymes (mitochondrial oxidative system) and/or the reduction of manganese superoxide dismutase (MnSOD, mitochondrial antioxidative system) can elevate the mitochondria-derived superoxide level (Robinson, 1998; Cadenas and Davies, 2000; Wallace, 2001; Murphy, 2009). Previous studies demonstrated that superoxide overproduction was primarily a consequence of the reduction in cellular mitochondrial complex I activity in patients with inherited mitochondrial complex I deficiency (Pitkanen and Robinson, 1996; Verkaart et al., 2007).

Our previous study has shown that CHF significantly reduces the protein expression and activity of mitochondrial complex enzymes (complex I, II, and III) and MnSOD in the NG including aortic baroreceptors (Tu et al., 2012). At the same time, the mitochondrial superoxide production in the NG from CHF rats was also increased. These results demonstrate the association of mitochondrial complex enzyme and MnSOD dysfunctions with elevation of the mitochondria-derived superoxide in NG neurons from CHF rats. To analyze the correlation between elevation of the mitochondrial-derived superoxide and reduced Na_v_ channel activation and cell excitability in baroreceptor neurons from CHF rats, adenoviral MnSOD (Ad.MnSOD) gene was transfected into the NG in our study (Tu et al., 2012). Our data demonstrated that transfection of the Ad.MnSOD gene into the NG restored the protein expression of MnSOD, reduced the mitochondria-derived superoxide, and reversed the expression and current density of Na_v_ channels and the cell excitability in aortic baroreceptor neurons from CHF rats. These data strongly suggest that elevation of the mitochondria-derived superoxide contributes to the reduced Na_v_ currents and the suppression of neuronal excitability in CHF aortic baroreceptor neurons. Although transfection of the Ad.MnSOD gene completely restored expression of the MnSOD protein, it did not normalize the mitochondrial-derived superoxide, and the protein expression and activation of Na_v_ channels in CHF aortic baroreceptor neurons toward the level seen in sham neurons. This inconsistency might be explained by following possibilities. Firstly, as mentioned above, both oxidative and antioxidative systems in the mitochondria could affect the mitochondria-derived superoxide. Recovering the ability of scavenging mitochondrial superoxide through Ad.MnSOD gene-induced overexpression of the MnSOD protein is insufficient to scavenge mitochondrial oxidative system-derived superoxide overproduction, because the function of mitochondrial complex enzymes is not improved in our study. Secondly, the cytosolic superoxide production system (such as NADPH oxidase components) also exists in the NG (Li and Zheng, 2011). Cytosolic superoxide and other endogenous factors might also mediate CHF-reduced Na_v_ channel activity in aortic baroreceptor neurons. Additionally, transfection of the Ad.MnSOD gene into the NG also significantly restored CHF-blunted arterial baroreflex function, measured by responses of blood pressure and heart rate to electrical stimulation of the aortic depressor nerve, and reflex changes of heart rate and cardiac sympathetic nerve activity in response to changes of arterial blood pressure (Zhang et al., 2014). These data clearly indicate that the mitochondria-derived superoxide overproduction in aortic baroreceptors contributes to the impairment of the arterial baroreflex sensitivity in CHF.

Overall, elevation of the endogenous mitochondria-derived superoxide is involved in the reduced Na_v_ current density and cell excitability in CHF aortic baroreceptor neurons through acutely decreasing the activation of Na_v_ channels and chronically reducing the protein expression of Na_v_ channels. Subsequently, the mitochondrial superoxide overproduction is further associated with the impairment of the arterial baroreflex function in the CHF state. Thus, far, there have been very few studies explored how superoxide modulates electrophysiological properties and expression of ion channels, especially no report focusing on the mitochondria-derived superoxide. Usually an inside-out or outside-out single-channel patch-clamp recording is used to measure the direct regulatory effect of superoxide on the single-channel open probability. However, loss of the mitochondria in a single-channel recording prevents us from measuring the direct effect of the mitochondria-derived superoxide on Na_v_ channels. Therefore, exploring the mechanisms underlying the acute modulation of the mitochondrial superoxide in Na_v_ channels will require the development of advanced techniques. As regards the mechanism(s) responsible for modulation of the mitochondrial superoxide in expression of Na_v_ channels, we discuss the details below.

## Regulatory effect of nuclear factor κB (NFκB) p65 on Na_v_ channel expression and cell excitability in aortic baroreceptor neurons in CHF

NFκB, a transcription factor, can regulate the expression of a number of genes involved in pathophysiological states, such as inflammatory disease and heart failure (Frantz et al., 2003; Valen, 2004; Israël, 2010; Van der Heiden et al., 2010). NFκB consists of five structurally related proteins, namely RelA (p65), RelB, c-Rel, p50, and p52. The p65/p50 heterodimer is the most abundant and widely expressed form of NFκB (Hoffmann and Baltimore, 2006). In the resting state, NFκB presents a silent form in the cytosol through tight binding to the specific inhibitor of κBα (IκBα) (Hoffmann and Baltimore, 2006; Israël, 2010). In response to multiple stimuli in pathophysiological conditions, IκB molecules are phosphorylated on Ser32 and Ser36 residues by activation of IKKβ kinases (Kabe et al., 2005; Häcker and Karin, 2006). The serine-phosphorylated IκB is ubiquitinated and degraded (Karin and Ben-Neriah, 2000; Kabe et al., 2005). As a consequence, NFκB binds to specific sites on DNA, and induces transcription of numerous target genes after NFκB is activated and translocated from the cytoplasm to the nucleus (Israël, 2010).

Although many studies have discovered the role of NFκB in target gene transcription, very few studies focus on the involvement of NFκB in regulating ion channel gene transcription. Shang et al. found that NFκB could directly bind to the SCN5A promoter, which was involved in angiotensin II/hydrogen peroxide-induced down-transcription of Na_v_1.5 channels (Shang et al., 2008). Therefore, NFκB may be involved in mitochondrial superoxide-lowered activation and expression of Na_v_ channels in baroreceptor neurons in the CHF state. Our recent study has shown that the IKK–IκB–NFκB signaling pathway exists in rat NG (Zhang et al., 2014). We also found that CHF increased the phosphorylated IKK, degraded the IκBα, and enhanced the phosphorylated NFκB p65 in the NG. More importantly, our study further confirmed that CHF enhanced the ability of NFκB p65 binding to the Na_v_ 1.7 promoter in the NG. These results provide the molecular evidence that the activation of NFκB p65 is associated with the change of Na_v_ 1.7 channels in nodose neurons from CHF rats. Additionally, to further clarify whether the activation of NFκB p65 is involved in CHF-decreased Na_v_ channels and neuronal excitability in baroreceptor neurons, NFκB p65 shRNA gene was *in vivo* transfected into CHF nodose neurons in our study. Transfection of NFκB p65 shRNA into the NG not only normalized the phosphorylated NFκB p65 protein, but also significantly increased the protein expression and current density of Na_v_ channels in CHF rats (Zhang et al., 2014), which indicates that NFκB p65 shRNA gene upregulates the protein expression of Na_v_ channels in the NG from CHF rats through inhibiting the phosphorylated NFκB p65 protein. Although current studies do not unveil the detail molecular mechanisms of how NFκB p65 shRNA increases the protein expression of Na_v_ channels in the NG from CHF rats, hyperactivation of NFκB is considered to downregulate the protein expression and current density of Na_v_ channels in nodose neurons from CHF rats, which is inconsistent with the common conception that NFκB binding with target gene activates the gene transcription (Ghosh et al., 1998; Valen et al., 2001; Frantz et al., 2003; McKenna and Wright, 2015). Furthermore, we also observed that transfection of NFκB p65 shRNA into the NG significantly improved the cell excitability of aortic baroreceptor neurons and resultant arterial baroreflex sensitivity in CHF rats (Zhang et al., 2014). These data suggest that activation of the NFκB signaling is involved in CHF-induced downregulation of the Na_v_ 1.7 channel, suppression of the aortic baroreceptor neuronal excitability, and impairment of the arterial baroreflex function.

As mentioned above, transfection of Ad.MnSOD gene into nodose neurons reduces CHF-induced elevation of the mitochondrial superoxide, reverses CHF-decreased activation of the Na_v_ channel and neuronal excitability in aortic baroreceptors (Tu et al., 2012), and improves CHF-impaired arterial baroreflex sensitivity (Zhang et al., 2014). Additionally, transfection of Ad.MnSOD gene also inhibits CHF-induced augmentation of the phosphorylated NFκB p65 in the NG tissue (Zhang et al., 2014). However, transfection of NFκB p65 shRNA does not affect the superoxide level in the NG from CHF rats (Zhang et al., 2014). From these data, we can deduce that inhibition of NFκB p65 improves the aortic baroreceptor function and arterial baroreflex sensitivity even if a high level of superoxide is preserved in the NG from CHF rats. Based on these results, we consider that superoxide overproduction-induced impairment of the aortic baroreceptor neuron and abnormality of the arterial baroreflex function in CHF rats is attributed to activation of the NFκB p65 in the NG. However, the exact mechanisms by which superoxide induces activation of the NFκB p65 in nodose neurons from CHF rats are yet unclear. In human endothelial cells, protein kinase C is involved in superoxide-induced NFκB activation (Ogata et al., 2000). It has also been reported that superoxide mediates interleukin-1β–induced IκBα degradation and consequent NFκB activation in bovine articular chondrocytes (Mendes et al., 2003). the IKK pathway also links superoxide with NFκB activation (Kabe et al., 2005). In our study, CHF increased the phosphorylated IKKβ, decreased the total IκBα, and enhanced the phosphorylated NFκB p65 in nodose neurons (Zhang et al., 2014). Therefore, it is possible that superoxide regulates activation of the NFκB p65 in nodose neurons from CHF rats through multiple signal-transduction pathways.

In our study, we only measured the modulatory role of superoxide-NFκB signaling in Na_v_ 1.7 channels in rat nodose neurons, because Na_v_ 1.7 channels are expressed in all nodose neurons (A-type and C-type nodose neurons) as a predominant Na_v_ channel α-subunit, but Na_v_ 1.8 and Na_v_ 1.9 channels are located only in C-type nodose neurons (Tu et al., 2010). Thus, far there is no report regarding NFκB binding sites on rat Na_v_ 1.8 and Na_v_ 1.9 channel promoters. However, we realize that future studies addressing the influence of superoxide-NFκB signaling on Na_v_ 1.8 and Na_v_ 1.9 channels in the CHF state are absolutely needed because Na_v_ 1.8 and Na_v_ 1.9 channels also have an important role in the baroreceptor function (Tu et al., 2010).

## Angiotensin II signaling pathway and reduced activation of the Na_v_ channels in the aortic baroreceptor neurons in CHF

Angiotensin (Ang) II has been recognized as a physiologically active peptide in multiple tissues including the NG (Allen et al., 1988a; Touyz, 2005). The physiological effects of Ang II including the maintenance of fluid homeostasis and blood pressure have been reported (Peach, 1977; Harris and Navar, 1985; Navar et al., 1996). Normally, Ang II receptors located on the cell membranes mediate these physiological actions of Ang II (Mehta and Griendling, 2007). It has been well-documented that circulating and tissue Ang II levels are increased in CHF patients and animal models of CHF (Liu et al., 2000; Roig et al., 2000; Cardin et al., 2003; van de Wal et al., 2006). Allen, et al. found that Ang II receptor binding sites exist in somata of nodose neurons and transport to terminals of nodose neurons (Allen et al., 1988a,b). Electrophysiological study also revealed that exogenous Ang II has the direct neuronal effect on nodose neurons through AT1R (Widdop et al., 1992). Moreover, some studies have demonstrated that Ang II down-regulates the arterial baroreflex function (Lee and Lumbers, 1981; Guo and Abboud, 1984; Garner et al., 1987).

Our recent study has also confirmed the involvement of Ang II in CHF-induced arterial baroreflex abnormality (Zhang et al., 2015). In our study, overexpression of the AT1R mRNA and protein and elevation of the local Ang II concentration in the NG from CHF rats were observed (Zhang et al., 2015). Additionally, local microinjection of losartan into the NG significantly improved CHF-attenuated arterial baroreflex sensitivity, whereas this drug did not change the arterial baroreflex sensitivity in sham rats. Furthermore, local application of exogenous Ang II in the NG from sham rats mimicked CHF to depress the arterial baroreflex function (Zhang et al., 2015). These results suggest that elevation of endogenous Ang II with AT1R overexpression in the NG contributes to the aortic baroreceptor dysfunction and subsequent down-regulation of the arterial baroreflex sensitivity in CHF rats, although a physiological level of endogenous Ang II does not affect the baroreceptor function and arterial baroreflex sensitivity. However, the local microinjection of losartan into the NG did not fully normalize the arterial baroreflex sensitivity in CHF rats toward the level seen in sham rats. We understand that the mechanism(s) responsible for CHF-blunted arterial baroreflex sensitivity are very complicated. The influence of endogenous Ang II on other cardiovascular reflex afferents [i.e., muscle reflex afferents, cardiac sympathetic afferents, and chemoreflex afferents (Khan and Sinoway, 2000; Li et al., 2006; Wang et al., 2007, 2008; Michelini et al., 2015)] and the interaction of these cardiovascular reflex afferents with baroreceptor afferents at the level of the NTS might also be potential factors to contribute to the arterial baroreflex dysfunction in the CHF state. Additionally, the effect of Ang II on central regions might be also accounted for the impairment of the arterial baroreflex function in CHF. Llewellyn et al. have reported that a high level of Ang II is detected in the plasma of CHF rats (Llewellyn et al., 2014). In particular, some studies have found that CHF induces overexpression of the AT1R in several central regions including rostral ventrolateral medulla, nucleus tractus solitarius, paraventricular nucleus, and subfornical organ, etc (Liu et al., 2006; Wang et al., 2008; Zheng et al., 2009; Zucker et al., 2009; Llewellyn et al., 2014). It has also been shown that Ang II also plays an important role in regulation of the cardiovascular system through these central regions (Casto and Phillips, 1984, 1986; Zhu et al., 2004; Liu et al., 2006; Wang et al., 2008; Zheng et al., 2009; Zucker et al., 2009; Llewellyn et al., 2014).

As mentioned above, reduced expression and activation of Na_v_ channels are involved in attenuation of the baroreceptor neuronal excitability and resultant impairment of the arterial baroreflex sensitivity in CHF rats. Therefore, it is possible that the Na_v_ channel is a potential target associated with the regulatory effect of Ang II on the aortic baroreceptor function. In isolated aortic baroreceptor neurons of sham rats, application of exogenous Ang II acutely inhibits Na_v_ currents, and pretreatment of losartan totally abolishes the inhibitory effect of Ang II on Na_v_ currents (Zhang et al., 2015), which supports the view that the acute inhibitory effect of Ang II on Na_v_ currents mediates Ang II-attenuated arterial baroreflex function. However, losartan alone did not change Na_v_ currents in isolated aortic baroreceptor neurons of CHF rats although this drug markedly improved the arterial baroreflex function in anesthetized CHF rats (Zhang et al., 2015). This discrepancy is explained by the fact that isolated aortic baroreceptor neurons of CHF rats loss the *in vivo* environment in which circulating (plasma) Ang II and paracrine release of Ang II from local tissue (the NG) are elevated in CHF rats (Llewellyn et al., 2014; Zhang et al., 2015). Based on these results, we consider that CHF-elevated endogenous Ang II with overexpression of AT1R inhibits activation of Na_v_ channels in aortic baroreceptor neurons and further contributes to attenuated arterial baroreflex sensitivity in CHF rats.

Previous study has shown that Ang II binds with AT1 receptors to cause superoxide production mainly through activation of NADPH oxidase (Touyz and Berry, 2002). However, there are no previous reports of how Ang II mediates CHF-induced hypoactivation of Na_v_ channels in aortic baroreceptors. In most cells including neurons, the mitochondria serve as the main source of superoxide production (Turrens, 2003). Ang II significantly elevated the mitochondria-derived superoxide in neurons (Yin et al., 2010; Case et al., 2013), leading to a series of downstream effects including modulation of the ion channel activation and neuronal firing rate (Zhu et al., 1999; Sun et al., 2005; Zimmerman et al., 2005; Yin et al., 2010). In particular, the mitochondria-derived superoxide overproduction mediates decreased Na_v_ currents and neuronal excitability in aortic baroreceptors from CHF rats (Tu et al., 2012). Based on these studies, it is reasonable to conclude that Ang II-induced inactivation of the Na_v_ channel might be linked to the mitochondrial superoxide overproduction in aortic baroreceptor neurons in CHF.

## Conclusion

**Figure 1:**
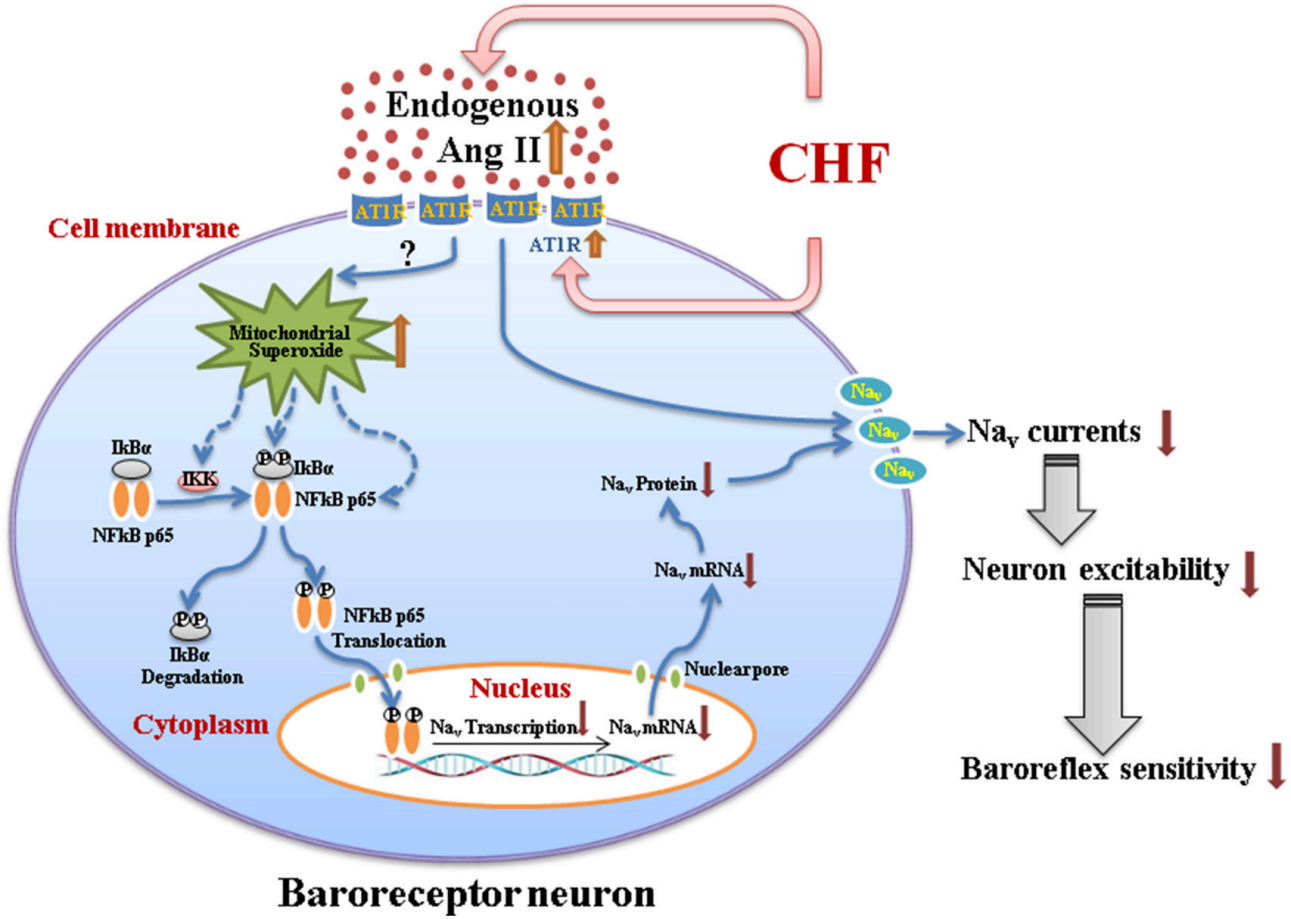
**A schematic diagram illustrating the mechanisms responsible for impairment of the arterial baroreceptor function in chronic heart failure**. CHF-elevated endogenous Ang II (plasma Ang II and tissue Ang II in the nodose ganglion) with overexpression of AT1R in baroreceptor neurons might induce the mitochondrial superoxide overproduction and the latter upregulates expression of the phosphorylated IKK, phosphorylated IκBα, and phosphorylated NFκB p65. The phosphorylated NFκB p65 translocates to the nucleus, and down-regulates the mRNA and protein expression of Na_v_ channels. Additionally, endogenous Ang II with AT1R also directly inhibits the activation of Na_v_ channels. As a consequence, reduced expression, and activation of Na_v_ channels in baroreceptor neurons contribute to the impairment of aortic baroreceptors and the arterial baroreflex dysfunction in the CHF state. Briefly, Ang II-superoxide-NFκB signaling pathway down-regulates the aortic baroreceptor function through influencing the expression and activation of Na_v_ channels in CHF. CHF, chronic heart failure; Ang II, Angiotensin II; AT1R, Angiotensin II type 1 receptor; Na_v_, voltage-gated sodium channel.

This review summarizes the mechanisms responsible for attenuated baroreceptor function and impaired arterial baroreflex in the CHF state. The information presented in this review suggests that Ang II-superoxide-NFκB signaling pathway down-regulates the neuronal excitability of aortic baroreceptors through influencing the expression and activation of Na_v_ channels on the cell transmembrane and subsequently causes the impairment of the arterial baroreflex in the CHF state (Figure 1). These new findings also reveal potential pharmacological targets for improving the arterial baroreflex function in the CHF state.

## Funding

This work was supported by the National Institutes of Health's National Heart, Lung, and Blood Institute grant R01 HL-098503 (to YL).

### Conflict of interest statement

The authors declare that the research was conducted in the absence of any commercial or financial relationships that could be construed as a potential conflict of interest.
